# Decoding the gene-disease associations in type 2 diabetes: A curated dataset for text mining-based classification

**DOI:** 10.1016/j.dib.2024.110418

**Published:** 2024-04-17

**Authors:** Sushrutha Raj, Sushmitha Raj, Vindhya Namdeo, Alok Srivastava

**Affiliations:** a Amity Institute of Integrative Sciences and Health, Amity University Haryana, Amity Education Valley, Gurgaon 122413, India; b Sri Innovation and Research Foundation, Ghaziabad 201009, India; c L V Prasad Eye Institute, Hyderabad 500034, Telangana, India

**Keywords:** Type 2 Diabetes genes, Double cross-validation, Meta-analysis, System modelling, Text mining, Machine learning, Text classifier, Type 2 Diabetes gene association

## Abstract

Type 2 Diabetes (T2D) exerts a substantial impact on mortality rates. According to 2023 statistics, more than half a billion individuals are experiencing the effects of T2D, making it one of the top 10 leading contributors to worldwide deaths. Multiple factors contribute to the onset of T2D, such as obesity, poor diet and lifestyle, the mutation in specific genes and many more. Among the various factors that contribute to the development of T2D, genetics is a pivotal aspect. Due to the significant influence of genes in the initiation and advancement of various phases of T2D, our focus lies on exploring the association between T2D and genes. In the present article, we have curated Standard disease gene association data which contains evidence or reference sentences which contain this disease gene association information, which is further classified into 4 classes: Yes, No, Ambiguous and X each pertaining to Positive, Negative, Ambiguous and Not related disease-gene associations respectively. For the purpose of this work, we downloaded T2D related abstracts from PubMed using EDirect and further pre-processed this abstract data to extract Reference Sentences Data. This data was later double-fold manually validated to compile this disease gene association data. The data produced in this article serves as reference data for the training text mining-based biological literature classifiers. Classifiers will further be used to predict classes of published literature, not just for T2D, but can also be expanded beyond to encompass a wide range of disease and their complications. The compilation of positively linked genes derived from these predictions can then be utilized for in-depth system-level analysis of T2D.

Specifications Table

**Table:** 

Subject	Health Informatics
Specific subject area	T2D genes, T2D gene classification, T2D gene association, Text classification
Type of data	Table; Raw, Processed, and Validated
Data collection	Raw data containing PubMed abstracts was collected using EDirect. Subsequently extracted the required fields: PubMed ID, Title, Date, Abstract Text.
Data source location	Institution: L V Prasad Eye Institute City/Province/Region: Hyderabad, Telangana Country: India
Data accessibility	Repository name: Mendeley Data Data identification number: 10.17632/23n5xfjhyt.2 Direct URL to data: https://data.mendeley.com/datasets/23n5xfjhyt/2

## 1 Value of the Data

• This disease-gene association (DGA) data holds immense value due to its meticulous curation and validation processes. The two-tier manual validation process enhances data quality, ensuring accuracy and reliability for a wide range of applications, from drug discovery and personalized medicine to informing public health strategies. By establishing robust associations between genes and T2D, this data offers a crucial foundation for advancing biomedical research. As a comprehensive resource, this data fosters collaboration across scientific disciplines, offering insights that can drive innovation, improves patient care, and ultimately contributes to a deeper understanding of T2D and its genetic complexities.
• This data caters to a broad spectrum of interests, promoting collaboration and knowledge dissemination across the research, clinical, industrial, and policy landscapes. Researchers across genetics, bioinformatics, Machine learning, Artificial Intelligence, Text Mining, Natural Language Processing and related fields can utilize the data to advance their investigations into T2D and genetic determinants. Clinicians can gain insights for more informed patient management, while pharmaceutical companies can identify potential gene targets for novel therapeutics. Public health organizations can leverage the data to shape evidence-based strategies for T2D prevention and management.
• Other researchers have the opportunity to use this rigorously validated DGA data in a multitude of ways. Researchers can leverage these associations to unravel the genetic underpinnings of T2D, identify potential therapeutic targets, and explore molecular mechanisms. Machine learning, Text Mining and network analysis techniques can be employed to uncover hidden patterns and interrelationships between genes driving novel discoveries. This data has the potential to function as a dependable standard for training, validating and replicating T2D Genetic association studies. This data can also be used to unveil valuable insights into the interconnected relationships between different subclasses of Diabetes Mellitus and its possible complications. By integrating these associations with additional omics data, scientists can gain a better understanding of the molecular pathways underlying T2D. Furthermore, the data may be leveraged to conduct a meta-analysis, which provides a comprehensive perspective on the genetics of T2D.

## 2 Background

Understanding the genes linked to T2D is vital due to the intricacies of the disease. While traditional methods such as Linkage Analysis, Positional Cloning, Sequencing and Pedigree Analysis are foundational [1], they prove to be time-consuming and expensive. Additionally, these methods can be labor-intensive and lack scalability, prompting a transition towards computational machine learning approaches [2,3]. However, this transition introduces a significant limitation, relying on existing databases for DGA data, resulting in errors like false positives and false negatives [4,5]. To address this, our study introduces a meticulously curated DGA dataset for T2D, compiled through a rigorous manual double-fold cross-validation process, ensuring robust validation.

The absence of a well-validated T2D-associated gene list in existing literature highlights our dataset's unique value. Unlike other resources, it serves as a crucial tool for meta-analysis, offering a comprehensive understanding of T2D nuances and pathways. Its importance extends beyond conventional analyses, particularly benefiting Biomedical Text Mining, aiding in Named Entity Recognition of Genes and Diseases. Moreover, the dataset includes association phrases, a valuable resource for researchers navigating complex biomedical literature. Serving as a cornerstone for abstract mining in the biomedical domain, this dataset becomes an unparalleled reference, enhancing the accuracy of machine learning techniques like classification and association mining. This dataset can be employed to extract disease-independent DGA associations, thereby enhancing its adaptability and utility across diverse disease contexts.

## 3 Data Description

The dataset [6] accompanying this data-in-brief article encompasses the subsequent files:

(1) Raw Abstract Data (Supplementary_File_1.xlsx): This file comprises raw abstract data pertaining to T2D. The extraction methodology is detailed in the materials sub-section 3.2.1, featuring four columns (“PMID”, “Title”, “Date”, and “Abstract text”) and 142529 rows.
(2) Gene Dictionary Data (Supplementary_File_2.xlsx): This file includes information on the Gene Dictionary utilized for extracting necessary DGA information from raw abstract data. The extraction process is expounded in the materials sub-section 3.2.2. It encompasses two sheets, namely “gene_to_appd_symbol_mapping” and “final_gene_dictionary”. The former contains the raw mapping of Gene Approved Symbol (“ApprovedSymbol”) to Gene Alias (“Alias Gene”) with associated categories in the “Alias Category” column, comprising three columns and 205488 rows. The latter, utilized for subsequent text processing of abstracts, contains the gene dictionary with 192799 entries. Table 1 below offers an excerpt of this file.

**Table 1:** Excerpt entries of the gene dictionary data.

Approved Symbol	Alias	Gene Alias Category
A1BG	alpha-1-B glycoprotein	Approved Name
A1BG-AS1	A1BG antisense RNA 1	Approved Name
A1CF	APOBEC1 complementation factor	Approved Name
A2M	alpha-2-macroglobulin	Approved Name
A2M-AS1	A2M antisense RNA 1	Approved Name
(3) Disease Dictionary Data (Supplementary_File_3.xlsx): This file contains information on the T2D Disease Dictionary utilized for extracting requisite DGA information from raw abstract data. The extraction details are outlined in the materials sub-section 3.2.3. It incorporates two sheets, namely “Dis_Synonyn_to_Acronym_Mapping” and “Final_Disease_Dictionary”. The former contains the mapping of “Disease Synonym” to “Acronym” with three columns and 819 rows. The latter, employed for subsequent text processing of abstracts, contains the disease dictionary with 59 entries. Table 2 below presents an excerpt of this file.

**Table 2:** Excerpt entries of the disease dictionary data.

S.No	Disease Synonym	Acronym
1	Adult Onset Diabetes	AOD
2	Adult-Onset Diabetes	AOD
3	Adult-Onset Diabetes Mellitus	AODM
4	Diabetes Mellitus Type 2	DMT2
5	Diabetes Mellitus Type II	DMT2
(4) Processed Data (Supplementary_File_4.xlsx): This file encompasses information on the final validated T2D disease-gene association data. The extraction details are delineated in the materials sub-section 3.2.4. It comprises 12 columns (“DB_ID”, “PMID”, “REF_SENTENCE” “DISEASE_NAME”, “GENE_NAME”, “VAL_REF_SENTENCE”, “VAL_DISEASE_NAME”, “VAL_GENE_NAME”, “GENE_NAME_APP”, “DGA_CLASS”, “DGA_PHRASE”, and “DGA_WEIGHT”) and 12346 rows.

## 4 Experimental Design, Materials and Methods

### 4.1 Experimental design

**Fig. 1:**
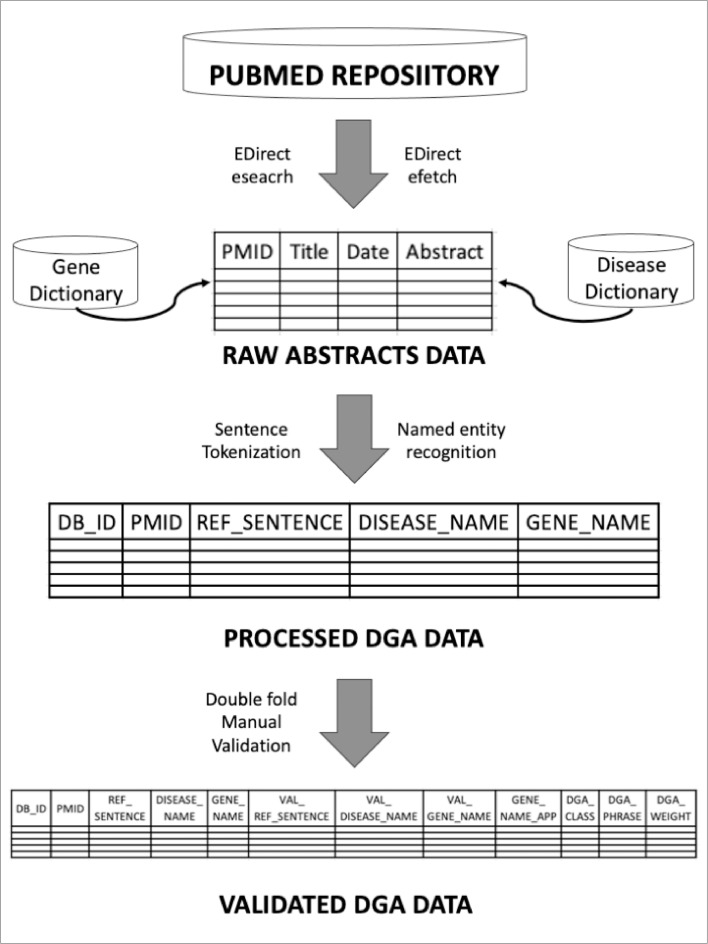
Workflow of the validation of DGA data. Sentence Tokenization and Named Entity Recognition are the text-processing steps employed to extract processed DGA data from raw abstracts data.

The workflow of the protocol utilized in this study is illustrated in Fig. 1, which consists of four major components as follows:

1. Extraction of “Raw Abstracts Data” from PubMed repository using EDirect
2. Pre-processing of Raw Abstracts Data to extract required Reference Sentences, “Processed DGA Data”
3. Double-fold Manual Validation of Pre-processed DGA Reference Sentences data
4. Calculation of DGA Weights using the “Validated DGA data”

Fig. 1 depicts the workflow of the protocol employed in the present study.

### 4.2 Materials

#### 4.2.1 Raw abstract data

The text data pertaining to T2D-related abstracts, necessary for the present study, was obtained by using the EDirect [7] platform. To extract the required data, the following filters were used: the search string “Type 2 Diabetes”, the availability of abstracts text, the designation of “English” as the language, the specification of “Human” as the species, and a time range limited to until December 15th, 2022. Table 3, provides a compact overview of the downloaded raw abstract data related to T2D.

**Table 3:** Summary of T2D-specific abstracts data.

Disease	Type 2 Diabetes
Source	E-direct
Date of download	15th Dec 2022
Query used	esearch -db pubmed -query “type 2 diabetes AND english [FILT] AND humans [MESH] AND has abstract [FILT]” | efetch -format medline > output_file.txt
Count	142529

The downloaded raw data had many fields which were unnecessary for the present study. We, therefore, extracted the required fields “PMID”, “Title, “Date” and “Abstract Text” for the purpose of the present study. This abstract data which was further processed and validated has been provided as Supplementary_File_1.xlsx.

#### 4.2.2 Gene dictionary

To create a complete dictionary for human protein-coding genes, we gathered detailed information extensively from the HGNC (HUGO Gene Nomenclature Committee) [8] on 24-08-2023. This comprehensive list included not just approved gene symbols, but also previous names, alternative symbols, and other names linked to that gene. Subsequently, the compiled list was employed to construct a comprehensive gene dictionary encompassing all gene name synonyms mapped to their approved gene symbols. The gene dictionary, thus created, was used in the Gene Name Entity Recognition (NER) for extraction of Reference Sentences. This Gene dictionary has been provided as Supplementary_File_2.xlsx.

#### 4.2.3 Disease dictionary

The present work includes the compilation of a Disease Dictionary specifically particular to T2D. The process for building this dictionary contained the curation of equivalent terms or synonyms for all the human protein-coding genes by reviewing of several databases. The databases used in this study included sources such as the Medical Subject Headings (MeSH) thesaurus [9], Ontobee [10], Unified Medical Language System (UMLS) [11], BioPortal [12], Human Disease Ontology (DO) [13], and International Classification of Diseases (ICD-11) [14]. The methodology used in constructing the Disease Dictionary was the consolidation of synonyms and their acronyms obtained through these sources. Subsequently, a diligent verification process was undertaken to ensure the accuracy of the information. As a result, the collection of 40 pairs of synonymous terms was obtained, and comprehensive information on these mappings can be found in Supplementary_File_3.xlsx.

Following that, the mappings were consolidated into a single list and any instances of duplication were removed in order to generate our final Disease Dictionary. This dictionary includes a total of 59 entries, which are documented in Supplementary_File_3.xlsx. The Disease Dictionary that is created serves as a point of reference for the NER component of this method. It is used to identify disease name entities that are mentioned in the prediction data.

## 5 Methods

### 5.1 Pre-processing of the raw abstract data

Subsequently, we processed the raw abstracts data to utilize text mining methodologies encompassing sentence tokenization and NER to systematically extract the reference sentences. Specifically, sentences within the abstract text wherein both the disease and gene names co-occur were extracted through a string-matching methodology using the Disease and Gene Dictionaries. This text pre-processing yielded in 12346 references sentences to be validated further. We further added a new column “DB_ID” as a unique ID for each entry to be used for traceback or reference This information of extracted reference sentences along with disease synonyms and gene names has been provided as Supplementary_File_4.xlsx. Table 4 below depicts an excerpt of a few entries from this processed data.

**Table 4:** Excerpt entries of the extracted abstracts reference sentence data.

DB_ID	PMID	REF_SENTENCE	DISEASE_NAME	GENE_NAME
DB_ID_2799	19877155	Thus, PSMD9 is a candidate T2D gene for the NIDDM2 locus.	T2D	PSMD9
DB_ID_2085	35242109	This study suggests CAPNS1 is a crucial gene in T2D hearts.	T2D	CAPNS1
DB_ID_602	30598999	No association was observed between AQP9 SNPs and T2DM risk.	T2DM	AQP9
DB_ID_663	16229747	In contrast, IPF1 is not a cause of type 2 diabetes in Caucasians.	Type 2 Diabetes	IPF1
DB_ID_1830	31759989	This study revealed an association of SIRT1 and WFS1 with T2D risk.	T2D	SIRT1, WFS1

### 5.2 Double-fold manual validation of processed data

The validation process for T2D associated genes employed a systematic approach double-fold cross-validation of reference sentences extracted using NER and String Matching. In this process, we read each reference sentence manually and validated whether or not this sentence has a DGA mentioned in it. If the sentence does contain DGA information, it is further classified into one of the association classes “Y”, “N” and “A” classes. That is, if the reference sentence showcased a positive association between the Reference Gene and a T2D synonym, it was designated “Y”. These sentences contained compelling evidence of their pertinence to T2D. If the reference sentence showcased a negative association between the Reference Gene and a T2D synonym, it was “N”. If the reference sentence showcased lacked definitive confirmation or refutation or in any sense has ambiguity, it was designated “A”. Otherwise it is classified into the “X” class. These sentences had no relevance to T2D or its association with the gene. This meticulous methodology ensures a systematic and rigorous approach to validating DGAs related to T2D, effectively categorising them into well-defined classes as per the context of the sentence.

While validating the entries we noted this validation information in the columns: "VAL_REF_SENTENCE", "VAL_DISEASE_NAME" and "VAL_GENE_NAME" and "DGA_CLASS" which have been described in the Table 5 below. After the DGA information was validated we mapped the validated Gene synonym mentioned in the reference sentence to its approved symbol in GENE_NAME_APP column, which was later further used for the calculation of the DGA weights for each block in DGA_WEIGHT column. We further mentioned the words or phrases implying the DGA for the respective entry in DGA_PHRASE column. The details of these columns added during the validation process have been provided in Table 5 below. This data can be used for Disease and Gene NER, DGA identification and DGA prioritisation. The validated data has been provided as Supplementary_File_4.xlsx.

**Table 5:** Description of columns added to the raw data.

S No	Column Name	Description
1	VAL_REF_SENTENCE	Validated reference sentence containing the DGA information
2	VAL_DISEASE_NAME	The validated T2D synonym mentioned in the reference sentence
3	VAL_GENE_NAME	The validated Gene synonym mentioned in the reference sentence
4	GENE_NAME_APP	Approved gene symbol of VAL_GENE_NAME column
5	DGA_CLASS	Assigned DGA class for the validated reference sentence
6	DGA_PHRASE	Words or phrases implying the DGA
7	DGA_WEIGHT	Disease gene association empirical weight

Table 6 provides a comprehensive summary of the subcategories within the “DGA_CLASS” column of the validated data. The “Association Class” column in the below table specifies the different classes of DGAs, while the “No of Entries” column indicates the number of entries corresponding to each association class. Additionally, the “No of genes” column reveals the count of genes associated with the entries in each association class. In total, there are 12,346 entries across three association classes, involving 1,015 total genes and 728 unique genes emphasizing the diversity and complexity of DGAs within the dataset.

**Table 6:** Summary of subcategories of DGA_CLASS in the validated data.

Association Class	No of Entries	No of genes
Y	2137	580
N	387	197
A	465	238
X	9357	-
Total	12346	1015
Unique Genes	-	728

### 5.3 Assigning DGA classes

After the DGA data was validated, it was observed that the same gene had multiple reference sentences and thus multiple DGA classes. We, therefore, put these multiple entries for the same gene into gene blocks that encompass diverse association classes. In these instances, empirical probabilities were calculated for each DGA class within a gene block, determining the DGA score for genes associated with T2D. This score information was added in the “DGA_WEIGHT” as mentioned in Table 5. Subsequently, the assignment of final DGA classes adopts a strategic approach maximizing the weight attained for each DGA class within a gene block. This approach ensures the definitive classification of these gene blocks. Supplementary_File_4.xlsx also provides comprehensive weight information for reference.

The extensively validated DGA information with scores and supportive evidence, establishes the dataset as a robust hallmark for the T2D DGA [6]. This dataset can be utilised to train machine learning models for precise DGA classification, with potential performance enhancements upon integration with similar datasets related to other diseases [15,16]. This way the disease-specific DGA classification can be upgraded to a disease-independent DGA classification protocol. Utilizing the allocated DGA classifications, it is feasible to extract genes exhibiting positive associations, which can further offer prospects for intricate system-level T2D modelling. Moreover, genes categorized as ambiguous in their associations furnish an opportunity for further investigation into their potential linkages with T2D pathology.

## Limitations

While our study represents a significant advancement in elucidating gene-disease associations in T2D, it is crucial to recognize inherent limitations. The dataset's size is constrained due to its focus on T2D, but integration with similar data from other diseases can enhance its resilience and disease-independence. Additionally, the accuracy of our findings relies on the quality and comprehensiveness of utilized materials, such as disease and gene dictionaries, which may undergo updates over time, introducing biases. Despite thorough manual validation, sometimes complex sentences may be subjective to interpretation biases that could affect the DGA extraction process. Nevertheless, our work offers valuable insights into T2D genetics, laying a foundation for future research in the field.

## Ethics Statement

Not applicable. The authors have read and follow the ethical requirements for publication in Data in Brief and confirm that this research did not require ethical approval since it does not concern humans or animals.

## CRediT authorship contribution statement

**Sushrutha Raj:** Methodology, Validation, Writing – original draft, Writing – review & editing, Supervision. **Sushmitha Raj:** Validation, Writing – original draft. **Vindhya Namdeo:** Validation, Writing – original draft. **Alok Srivastava:** Conceptualization, Methodology, Writing – original draft, Writing – review & editing, Supervision, Project administration, Funding acquisition.

## Data Availability

Reference Dataset for Text Mining Type 2 Diabetes Candidate Genes (Reference data) (Mendeley Data)
